# BRD4 Inhibitor GNE-987 Exerts Anticancer Effects by Targeting Super-Enhancer-Related Gene LYL1 in Acute Myeloid Leukemia

**DOI:** 10.1155/2022/7912484

**Published:** 2022-08-01

**Authors:** Xu Sang, Yongping Zhang, Fang Fang, Li Gao, Yanfang Tao, Xiaolu Li, Zimu Zhang, Jianwei Wang, Yuanyuan Tian, Zhiheng Li, Di Yao, Yumeng Wu, Xinran Chu, Kunlong Zhang, Li Ma, Lihui Lu, Yanling Chen, Juanjuan Yu, Ran Zhuo, Shuiyan Wu, Zhen Zhang, Jian Pan, Shaoyan Hu

**Affiliations:** ^1^Department of Hematology, Children's Hospital of Soochow University, Suzhou 215003, China; ^2^Department of Pediatrics, The First Affiliated Hospital of Bengbu Medical College, Bengbu 233004, China; ^3^Institute of Pediatric Research, Children's Hospital of Soochow University, Suzhou 215003, China; ^4^Intensive Care Unit, Children's Hospital of Soochow University, Suzhou 215003, China

## Abstract

**Background:**

AML (acute myeloid leukemia) is a common hematological malignancy in children with poor treatment effects and poor prognosis. Recent studies have shown that as a novel BRD4 (bromodomain containing 4) PROTACs (proteolysis targeting chimeras) degrader, GNE-987 can slow down the growth of various tumors and increase apoptosis, with promising clinical prospects. However, the function and molecular mechanism of GNE-987 in AML remain unclear. This study is aimed at investigating the therapeutic effect of GNE-987 on AML and its underlying mechanism.

**Methods:**

The association between BRD4 and AML was assessed by studying public databases. After GNE-987 was added to AML cells, cell proliferation slowed down, the cycle was disturbed, and apoptosis increased. Western blotting was used to detect BRD2 (bromodomain containing 2), BRD3 (bromodomain containing 3), BRD4, and PARP (poly ADP-ribose polymerase) proteins. The effect of GNE-987 on AML cells was analyzed in vivo. RNA-seq (RNA sequencing) and ChIP-seq (chromatin immunoprecipitation sequencing) validated the function and molecular pathways of GNE-987 in processing AML.

**Results:**

BRD4 expression was significantly elevated in pediatric AML samples compared with healthy donors. GNE-987 inhibited AML cell proliferation by inhibiting the cell cycle and inducing apoptosis. BRD2, BRD3, and BRD4 were consistent with decreased VHL (Von Hippel Lindau) expression in AML cells. In an AML xenograft model, GNE-987 significantly reduced the hepatosplenic infiltration of leukemia cells and increased the mouse survival time. Based on analysis of RNA-seq and ChIP-seq analyses, GNE-987 could target multiple SE- (super-enhancer-) related genes, including LYL1 (lymphoblastic leukemia 1), to inhibit AML.

**Conclusions:**

GNE-987 had strong antitumor activity in AML. GNE-987 could effectively inhibit the expression of SE-related oncogenes including LYL1 in AML. Our results suggested that GNE-987 had broad prospects in the treatment of AML.

## 1. Background

AML (acute myeloid leukemia) is the most common hematological malignancy in adults and ranks second in childhood hematological malignancies [1, 2]. It is a serious threat to children's physical and mental health. The pathogenesis of acute myeloid leukemia is unknown, and it is mostly believed to be related to abnormal epigenetic events caused by DNA or chromatin modification [3]. The epigenetic target screening using the shRNA (short-hairpin RNA) library and genome-wide CRISPR library proves that the BET (bromodomain and extraterminal) protein family member BRD4 (bromodomain containing 4) is the most important member of the BET family of histone reading proteins, which is essential to maintain AML [4, 5].

The acetylated lysine residues in histone H4 can bind to BETP (bromodomain and extraterminal protein), which provides the assembly of multimolecular superenhancer complexes [6, 7]. The HLH (helix loop helix) TF (transcription factor) family contains key regulators of lymphocyte development and maturation, such as Tal1 (T-cell acute lymphoblastic leukemia 1)/SCL (stem cell leukemia)/TCL5 (T-cell leukemia/lymphoma 5) [8]. The other two basic HLH (bHLH) TFs, LYLl and Tal2 (T-cell acute lymphoblastic leukemia 2), are closely related to Tal1 in structure. Studies have shown that LYL1 is considered a super-enhancer-associated gene that causes AML [9, 10]. MYC (myelocytomatosis viral oncogene) is one of the key oncogenes that rely on transcription mediated by the hyperenhancer complex containing BETP [11, 12]. LYL1 and MYC lack pockets that can be directly targeted by small molecules, and directly targeting them is difficult. Therefore, a lot of energy has been focused on indirect targeting strategies.

Some of the previous BRD4 inhibitors, such as JQ1 and I-BET, by disrupting the binding of BETP to acetylated histones, provide a way to target transcription by disrupting the “superenhancer” transcription complex MYC [11, 12]. However, these drugs cannot inhibit transcription, leading to drug resistance. PROTACs (proteolysis targeting chimeras) are bifunctional molecules that promote protein target degradation rather than inhibit activity as a therapeutic strategy. These molecules contain a motif (peptide or small molecule) that binds a protein target connected by a chemical linker to a motif that binds to E3-ubiquitin ligase. This allows E3-ubiquitin protein ligase to recruit to the protein target, selectively become the target of ubiquitination, and promote its degradation through the cell's endogenous proteasome degradation mechanism. PROTAC activity requires the formation of a ternary complex similar to a three-body structure in the 1 : 1 : 1 subunit stoichiometry, which contains the target protein, PROTAC, and E3-ubiquitinated ligase [6, 13]. Traditional inhibitor molecules require a 1 : 1 stoichiometry to inhibit a single protein target molecule, and PROTAC can play a substoichiometric role using one PROTAC molecule to promote the degradation of multiple copies of the target protein because PROTAC is released after protein degradation. This allows for lower dosing concentrations and a larger therapeutic window and reduces the need to maintain high intracellular compound concentrations. The effective time of targeting degraded proteins is also shorter, and the weaker and lower affinity region can be used as the target site. Therefore, it can target the previously difficult-to-degrade protein [14].

As a new type of BRD4 PROTAC degradation agent, GNE-987 is a ternary complex formed between BRD4B1 and BRD4B2 (BRD4 bromodomains 1 and 2) and VHL E3-ubiquitin ligase. BRD4 is an effective drug target affected by various cancers, whereas VHL is usually recruited by PROTACs to degrade various targets in vitro and in vivo. GNE-987 has previously been proven to be a more effective in vitro degradation product of BRD4 than standard PROTACs MZ1 and ARV-825. The half-life measurement results of the ternary complex based on SPR (surface plasmon resonance) show that the BRD4B1 ternary complex is more stable than the ternary complex containing BRD4B2 [15]. The binding of GNE-987 to the target can improve the stability and pharmacokinetics in vivo and effectively increase the degradation of BRD4 and the killing of tumor cells by GNE-987 [16]. GNE-987 inhibits proliferation and induces cell apoptosis more effectively than traditional BRD4 inhibitors and has a longer-lasting drug effect, possibly through the rapid and durable degradation of BRD4 and inhibition of downstream targets. However, at present, no study has focused on GNE-987 in AML. Therefore, we examined the antitumor activity of GNE-987 on BRD4 in AML and confirmed that it downregulated the expression of many superenhancers and related oncogenes, such as LYL1, to determine an effective strategy for the treatment of children with AML.

## 2. Materials and Methods

### 2.1. Samples

To determine the potential utility of targeting BRD4 in the treatment of AML, we analyzed the expression of BRD4 based on public RNA-seq (RNA sequencing) data in AML samples. Standardized gene expression data were used to assess the prognostic significance of BRD4 and the correlation between the two, and the overall survival rate of patients with BRD4 and AML patients was also analyzed.

### 2.2. Cell Culture

Human leukemia cell lines NB4, Kasumi-1, HL-60, MV4-11, and K562 and mouse leukemia cell line P388-D1 were all from the Chinese Academy of Sciences Cell Bank. They were all verified by short tandem repeat analysis in 2019 and 2020. Cells were cultured in RPMI1640 (Roswell Park Memorial Institute 1640) medium containing 10% FBS (fetal bovine serum) (Thermo Fisher Scientific, MA, USA) and penicillin and streptomycin (Millipore, Billerica, MA, USA) at 37°C and 5% CO_2_ in a humidified incubator and routinely tested for mycoplasma.

### 2.3. Cell Viability Determination

GNE-987 was dissolved in 100% DMSO (dimethyl sulfoxide), the stock solution had a concentration of 10 mmol, and it was placed in a refrigerator at -80°C. AML cells were planted in a 96-well cell culture plate with a cell density of 2 × 10^4^ in each well and were treated with GNE-987 with different concentration gradients. The primary leukemia cells were separated from the bone marrow of children by Ficoll-Hypaque centrifugation and then planted in a 96-well plate with a density of 1 × 10^5^ cells in the culture medium. Cells treated with 0.05% DMSO in a complete medium without GNE-987 were used as controls. After 24 hours of drug treatment, according to the manufacturer's instructions, cell viability was determined by the CCK8 (Cell Counting Kit 8) assay (Dojindo Molecular Technologies, Tokyo, Japan). Each concentration was in triplicate, repeated in at least three independent experiments. Graph Prism software 8.3.0 (GraphPad Software Inc., San Diego, CA, USA) was used to calculate the half-maximum inhibitory concentration (IC50) of GNE-987.

### 2.4. Soft Agar Clone Formation Analysis

Agarose and autoclave (1.2% and 0.7%) were prepared and placed in a 55°C water bath. 2x RPMI1640 medium containing 20% FBS, 2x penicillin, and streptomycin was prepared and filtered out with a 0.2-micron filter bacteria. For the lower layer gel, 1.2% agarose gel was mixed with 2x medium 1 : 1, added to a 6-well plate 1.5 ml per well, and solidified at room temperature. For the cell count, AML cells treated with GNE-987 at different concentration gradients were washed with PBS (phosphate-buffered saline), mixed and diluted with new medium, and adjusted to 5 × 10^3^/ml with 100 *μ*l of cell suspension in each well. For the upper glue, 0.7% agarose gel was mixed with 2x medium 1 : 1, and 100 *μ*l of cell suspension was added. After mixing wells, 1.5 ml was added to each well. The cells were placed in a 37°C CO_2_ incubator, the culture medium was added every three days, and the cells were harvested after approximately 3 weeks. The number of cells treated with different concentrations of GNE-987 was counted, compared, and finally analyzed to calculate the rate of monoclonal formation.

### 2.5. Preparation and Infection of Lentivirus

shRNA (short-hairpin RNA) targeting VHL (GGAGCCTAGTCAAGCCTGAGA CATCCGTTGATGTGCAATGCG) was constructed in the pLKO.1 lentiviral vector. The CDS region of the VHL gene was searched in PubMed, synthesized, and constructed into the PLVX-EF1a-puro vector. Short-hairpin RNA (shRNA) targeting LYL1 (Table 1) was constructed in the pLKO.1-puro lentiviral vector (IGE Biotechnology Ltd., Guangzhou, China). When preparing lentivirus, envelope plasmid and packaging plasmid were purchased from Addgene (pMD2.G: #12,259; psPAX2: #12,260; Cambridge, MA, USA). pMD2.G, psPAX2, and the transfer plasmid with polyethyleneimine were cotransfected into 293FT cells (linear MW 25000 Da, 5 mg/ml, pH 7.0) (cat. No. 23966-1; Polysciences, Warrington, PA, USA) according to the manufacturer's instructions. After 6 h, the medium was completely replaced with fresh medium. The virus supernatant was collected 48 h after transfection and filtered with a 0.22 *μ*m filter. Then, AML cells were infected with lentivirus for 24 hours in the presence of 10 *μ*g/ml polyene (Sigma-Aldrich). Stable cell lines were screened with puromycin (Sigma-Aldrich).

### 2.6. RNA Preparation and Real-Time PCR (Polymerase Chain Reaction) Expression Analysis

Total RNA was extracted from cell pellets using the TRIzol® reagent (Invitrogen, CA, USA), according to the manufacturer's protocol. For cDNA synthesis, 1 *μ*g of total RNA was converted to cDNA using a high-capacity cDNA reverse transcription kit (Applied Biosystems, CA, USA). Quantitative real-time PCR analysis was carried out using LightCycler® 480 SYBR Green I Master Mix (cat. No. 04707516001; Roche, Penzberg, Germany) with a LightCycler 480 Real-Time System (Roche), according to the manufacturer's protocol. mRNA expression levels were calculated using the Ct method with GAPDH (glyceraldehyde 3-phosphate dehydrogenase) expression as an internal reference. Primer sequences are listed in Table 2.

### 2.7. Cell Cycle Analysis

At 24 hours after adding different concentrations of GNE-987 to the AML cell line, the cell line was trypsinized, washed, and fixed in 70% ethanol at 4°C overnight. Then, the cells were washed with cold PBS, resuspended in 0.5 ml of PI/RNase staining fermentation broth (cat. No. 550825; BD Pharmingen™, San Diego, CA, USA), and then incubated at room temperature for 15 min. Flow cytometry was performed using the Beckman Gallios™ Flow Cytometer (Beckman, Krefeld, Germany), and the cell cycle was analyzed using Multicycle AV DNA analysis software (Verity Software House, Topsham, ME, USA).

### 2.8. Cell Apoptosis Analysis

Different concentrations of GNE-987 were added to the cell line and collected after 24 h, centrifuged at 2000 rpm for 3 min. Then, the supernatant was removed, and the remains were washed once with cold PBS and centrifuged at 4000 rpm for 3 min. After taking the supernatant, they were suspended in a 1x binding buffer. The fluorescein isothiocyanate-Annexin V apoptosis kit and PI solution staining (cat. No. 556420; BD Biosciences, Franklin Lakes, NJ, USA) were used as per the manufacturer's instructions. The cell counting method was adopted to analyze cell apoptosis (Beckman Gallios™ Flow Cytometer; Beckman).

### 2.9. Western Blotting Analysis

The following antibodies were used for Western blotting analysis: BRD2 (cat. No. 5848 s; 1 : 1000; Cell Signaling Technology, Boston, MA, USA), BRD3 (cat. No. 11859-1-AP; 1 : 1000; Proteintech, Chicago, IL, USA), BRD4 (cat. No. 13440 s; 1 : 1000; Cell Signaling Technology), VHL (cat. No. 68547 s; 1 : 1000; Cell Signaling Technology), LYL1 (cat. No. sc-374164; 1 : 1000; Santa Cruz Biotechnology), and PARP (cat. No. 9542; 1 : 1000; Cell Signaling Technology) with glyceraldehyde 3-phosphate dehydrogenase (GAPDH) (cat. No. MA3374; 1 : 1000; Millipore) as a reference protein. Peroxidase-conjugated AffiniPure goat anti-rabbit IgG (H+L) (cat. 111-035-003; 1 : 5000) and goat anti-mouse IgG (H+L) (cat. No. 115-035-003; 1 : 5000) were purchased from Jackson Immuno Research Laboratories, Inc. (West Grove, PA, USA). To define the role of the proteasome, MG132 (cat. No. 474787, Sigma-Aldrich, St. Louis, MO, USA) inhibited the proteasome activity. After 24 h of treatment with different concentrations of GNE-987, the cells were collected, and the BRD2, BRD3, BRD4, PARP, VHL, LYL1, and GAPDH proteins were determined by Western blotting analysis.

### 2.10. Study of the Antitumor Effect of GNE-987 In Vivo

In this study, all experimental animal procedures in this study were approved and licensed by the Animal Care and Use Committee of Children's Hospital of Soochow University (CAMSU-AP#: JP-2018-1). SPF-grade BALB/c mice were obtained from Linghang Biotechnology Co., Ltd. (Shanghai, China). Five-week-old female mice (*n* = 5 in each group) were injected with 3 × 10^5^ P388-D1 cells via the tail vein. Two days after the injection of cells, each mouse was injected with luciferase into the abdominal cavity and immediately anesthetized with isoflurane gas. Then, each group of mice was imaged using the NightOWL In Vivo Imaging System (Berthold, Germany). After the tumor fluorescence signal appeared (day 2), the experimental group was injected intraperitoneally with 0.5 mg/kg GNE-987, and the control group was injected intraperitoneally with GNE-987 (5%®HS15) once a day, nine times in total (days 2~10). Then, we continued to use the NightOWL In Vivo Imaging System to image each group of mice on days 4, 7, and 10. The mice were weighed daily, and fur color and mobility were observed. The liver, spleen, kidneys, and intestines of the experimental group and control group mice were collected, and organ size was observed and weighed. Each organ specimen was subjected to immunohistochemistry and HE (hematoxylin and eosin) staining. The primary antibody against BRD4 (cat. No. 13440 s; 1 : 1000; Cell Signaling Technology), cleaved-caspase 3 (cat. No. GB11009-1, 1 : 300, Servicebio, Boston, MA, USA), and Ki67 (cat. No. ab15580, 1 : 300, Abcam, Cambridge, UK) was used according to the manufacturer's recommendations.

### 2.11. RNA-Seq and Data Processing

RNA-seq was conducted according to the protocols suggested by Novogene Bioinformatics Technology Co., Ltd. (Beijing, China). First, the total RNA was reverse transcribed into cDNA to construct a library, and then, the cDNA library was then sequenced. The original reads were filtered, and the clean reads were mapped according to HISAT (Hierarchical Indexing for Spliced Alignment of Transcripts). Then, the gene expression level was calculated (as the fragment mapped per million reads per kilobase exon model). Using DESeq2 analysis, differentially expressed genes were identified (*P* < 0.05 and fold change > 2 or fold change < 0.5). For enrichment analysis, differentially expressed genes were analyzed using the GSEA software (UC San Diego and Broad Institute).

### 2.12. Chromatin Immunoprecipitation Sequencing (ChIP-seq)

Furthermore, 3-5 × 10^7^ cells were cross-linked with 1% formaldehyde for 10 minutes and neutralized with 1.25 M glycine at room temperature for 5 minutes. Bioruptor (Diagenode, Liège, Belgium) was used to collect, lyse, and sonicate fixed cells. Sonicated chromatin was incubated with an anti-histone H3 (acetyl K27) antibody (cat. No. ab4729; Abcam, Cambridge, UK) overnight at 4°C. DNA was eluted and purified using a QIAquick PCR purification kit (cat. No. 208106; Qiagen, Hilden, Germany). The samples were sequenced on the NovaSeq 6000 platform (Novogene Bioinformatics Technology Co., Ltd. Beijing, China) and a BGISEQ 2000 platform (Beijing Genomics Institute, Shenzhen, China). Raw data of ChIP-seq H3K27ac analysis were aligned to the reference genome (UCSC hg38) using Bowtie2 (v 2.3.5) [17], with alignment parameters -p 4 -q -x. Peaks were identified using MACS2 (v2.0.9) [18], with parameters -g hs -n test -B -q 0.01. The bedGraph files generated by MACS2 were converted to bigwig files using the UCSC bedGraphToBigWig tool, and bigwig files were then visualized by Integrative Genomics Viewer (IGV) [19]. Superenhancers were then identified using the ROSE (Rank Order of Superenhancers) method [20, 21], with parameters -s 12500 -t 2000.

### 2.13. Statistical Analysis

All experiments were conducted at least three times independently. Statistical analysis was carried out using IBM SPSS Statistics for Windows, version 21.0 (IBM Corp., NY, USA). Survival analysis was performed by Kaplan-Meier estimates with log-rank tests. Student's *t*-test was used to compare the percentage of apoptosis, BRD4 mRNA level, and cell viability. Normally distributed measurement data were expressed as the mean ± standard deviation. The *t*-test was used to compare the differences between the two groups. Nonnormally distributed data were expressed in quartiles (usually the median plus the range) and were compared using the Mann-Whitney *U* test. All experiments were two-tailed, and *P* < 0.05 was considered significant.

## 3. Results

### 3.1. BRD4 Is Overexpressed in Patients with AML and Is Associated with Poor Prognosis

**Figure 1:**
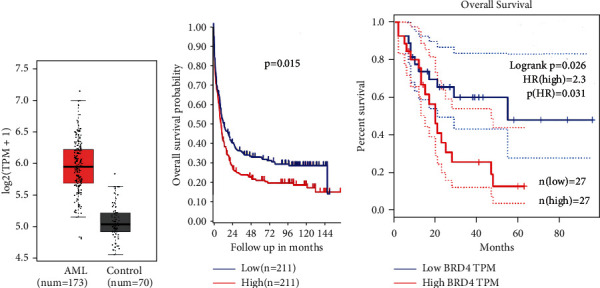
BRD4 is a potentially good target for AML. (a) Expression of BRD4 with AML and normal controls according to the GEPIA database (http://gepia.cancer-pku.cn/index.html). (b) The expected overall survival rate of high BRD4 expression with AML is lower than that of patients with low BRD4 expression, according to the R2 database (https://hgserver1.amc.nl/cgi-bin/r2/main.cgi). (c) The expected overall survival rate of high BRD4 expression in patients with AML is lower than that of patients with low BRD4 expression, according to the GEPIA database (http://gepia.cancer-pku.cn/index.html).

Compared with the normal population, BRD4 expression in patients with AML increased significantly according to the GEPIA (Gene Expression Profiling Interactive Analysis) database (http://gepia.cancer-pku.cn/index.html, Figure 1(a)). The expected overall survival rate of high BRD4 expression in patients with AML was lower than that of patients with low expression, according to the R2 database (https://hgserver1.amc.nl/cgi-bin/r2/main.cgi, Figure 1(b)) and the GEPIA database (http://gepia.cancer-pku.cn/index.html, Figure 1(c)). These results suggested that BRD4 would become a potential therapeutic target for pediatric AML.

### 3.2. GNE-987 Causes the Death of the AML Cell Lines and Inhibits Its Growth

**Figure 2:**
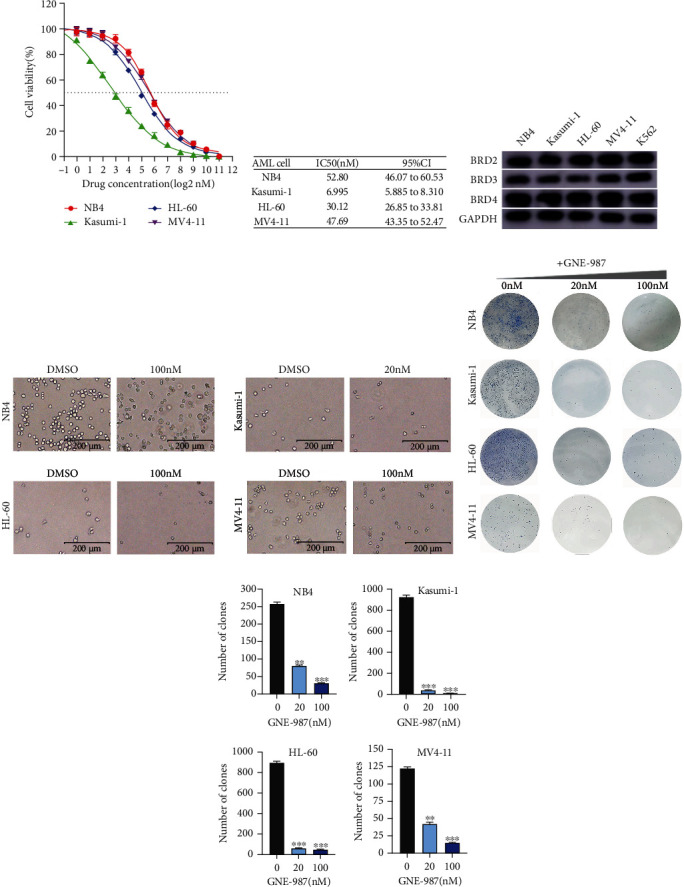
GNE-987 can cause the death of AML cell lines and inhibit their growth. (a) Cell viability curve after adding different concentration gradients of GNE-987 to NB4, Kasumi-1, HL-60, and MV4-11 cell lines. (b) IC50 values of GNE-987 with different concentration gradients were added to AML cells. (c) Basal BET protein level analysis in myeloid cell lines NB4, Kasumi-1, HL-60, MV4-11, and K562. (d) Fluorescence microscope photos of AML cell lines NB4, Kasumi-1, HL-60, and MV4-11 24 hours after adding GNE-987. (e) Number of AML cell clones added with different concentrations of GNE-987. (f) Statistical histogram of the number of AML cell clones added with different concentrations of GNE-987.

The cell viability curve after adding different concentration gradients of GNE-987 to AML cells is shown in Figure 2(a). The IC50 and 95% CI of GNE-987 in different AML cells are shown in Figure 2(b). The half-inhibitory concentration value was low at the nmol level. The BET family members are universally expressed in myeloid leukemia cell lines (Figure 2(c)). Fluorescence microscopy showed that the vast majority of AML cells died 24 hours after the addition of GNE-987 (Figure 2(d)). After adding GNE-987 to AML cells, cell growth was slower compared with the group without GNE-987, the number of clones decreased, and the difference was significant (Figures 2(e) and 2(f)).

### 3.3. GNE-987 Blocked Cell Cycle and Promoted Apoptosis of AML Cell Lines

**Figure 3:**
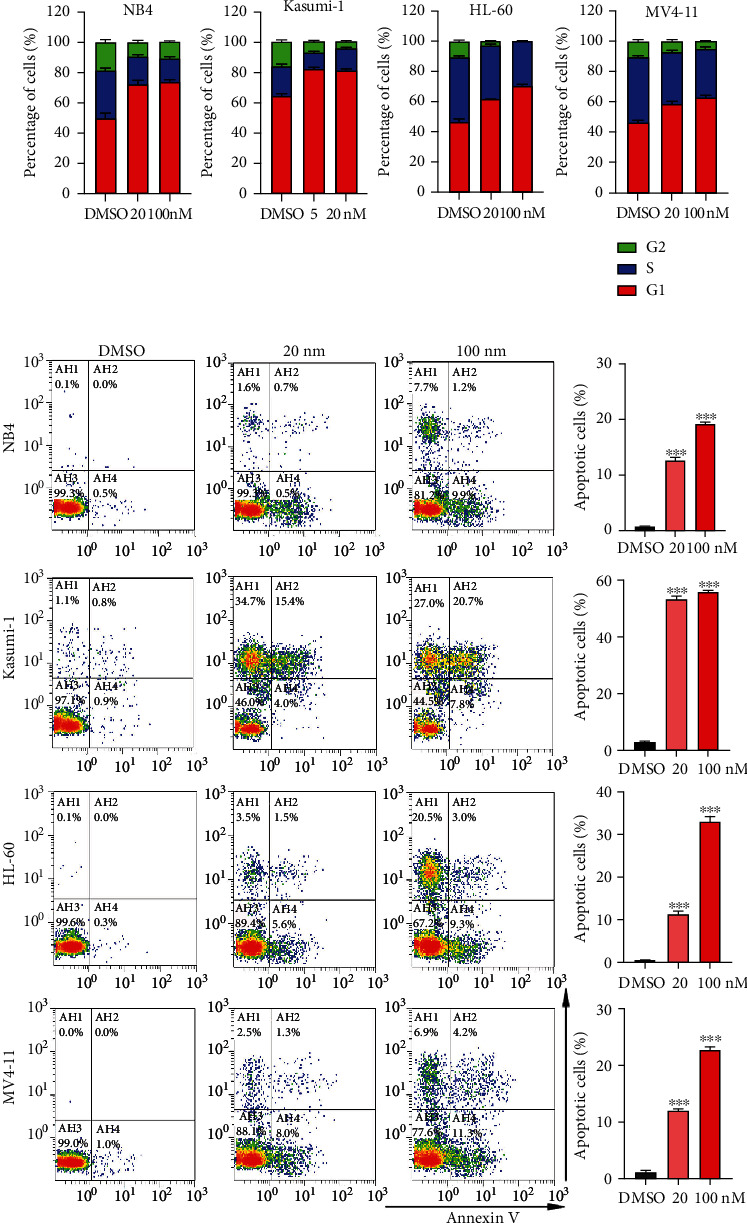
GNE-987 blocked the cell cycle and promoted the apoptosis of AML cell lines. (a) PI-labeled cell cycles of NB4, Kasumi-1, HL-60, and MV4-11 cells were analyzed after treatment with DMSO or different concentrations of GNE-987 for 24 h. AML cells were distributed in the G1/S phase, and the cell population in the G1 phase increased dramatically after treatment with GNE-987. (b) Annexin V and PI-labeled cell apoptosis of AML cells were analyzed by flow cytometry after treatment with DMSO or different concentrations of GNE-987 treatment for 24 h. The apoptotic rates of AML cells were significantly increased after GNE-987 treatment.

By influencing the cell cycle and promoting cell apoptosis, GNE-987 has higher cytotoxicity in AML. We detected cell cycle defects by PI staining. Most AML cells were distributed in the G1/S phase, but after 24 hours of treatment with GNE-987, the proportion of cells in the G1 phase increased significantly (Figure 3(a)). In addition, cell treatment with GNE-987 was for 24 hours, which increased the apoptotic rate of AML cell lines (Figure 3(b)).

### 3.4. GNE-987 Causes Degradation of BET Protein in AML Cell Lines

**Figure 4:**
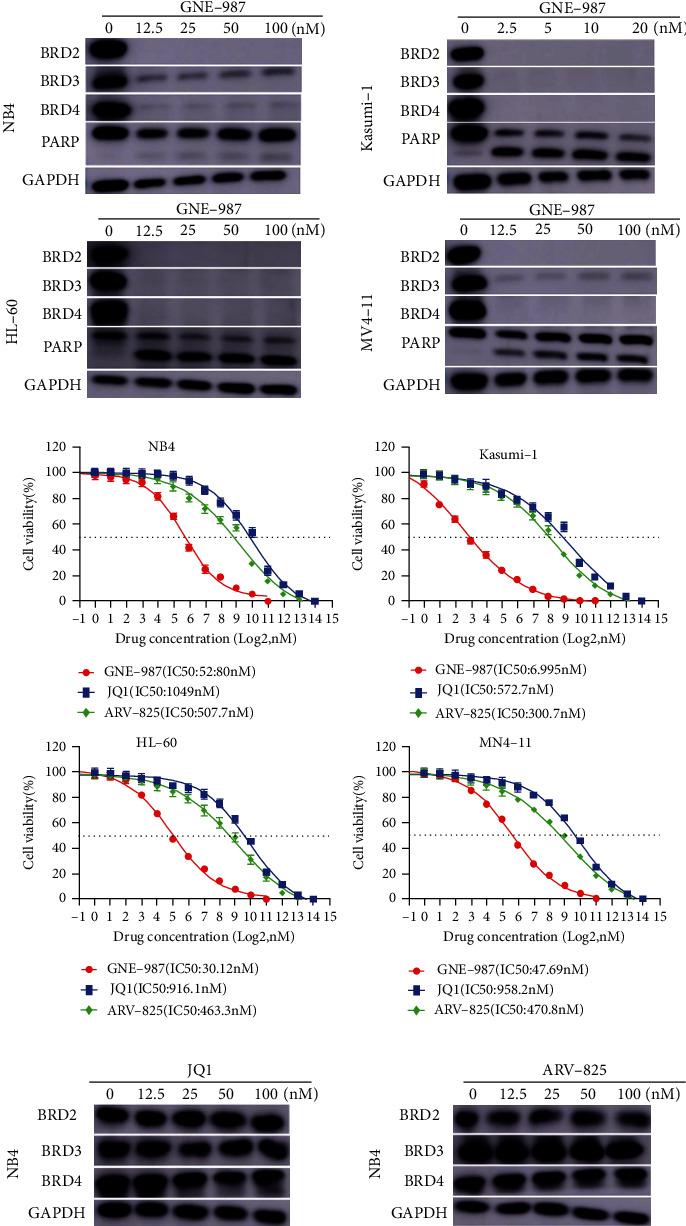
GNE-987 inhibits the expression of BET protein in AML cells and increases the expression of PARP protein, and the effect is far better than those of JQ1 and ARV-825. (a) Western blotting analysis showed that GNE-987 induced BET protein degradation, and PARP increases in NB4, Kasumi-1, HL-60, and MV4-11 cell lines. (b) Drug sensitivity assay of NB4, Kasumi-1, HL-60, and MV4-11 cell lines after treatment with gradient concentrations of GNE-987, JQ1, and ARV-825 for 24 h. (c) After adding JQ1 and ARV-825 at the highest concentration of 100 nM, the degradation efficiency of BET protein is much lower than that of GNE-987.

GNE-987 was designed with PROTAC technology to selectively degrade target proteins through the ubiquitin proteasome system. Therefore, we analyzed the BET protein expression of AML cell lines after treatment with five different concentrations of GNE-987 for 24 hours. Western blotting analysis showed that GNE-987 induced the degradation of BET protein and increased PARP (Figure 4(a)). BRD4 protein was almost completely degraded in the AML cell lines treated with GNE-987. In addition to BRD4, GNE-987 also reduced the expression levels of BRD2 and BRD3 proteins. These data indicated that GNE-987 downregulated BET protein expression in AML cells. We also compared the drug sensitivity test of GNE-987, JQ1, and ARV-825 gradient treatment of AML cell lines for 24 hours (Figure 4(b)). The effect of GNE-987 was much higher than that of JQ1 and ARV-825. We added JQ1 and ARV-825 to NB4 cells 24 hours later to observe the degradation efficiency of BET protein. As shown in Figure 4(c), after adding JQ1 and ARV-825 at the highest concentration of 100 nM, the BET protein was almost not degraded.

### 3.5. VHL Is the Key E3 Enzyme for the Function of GNE-987, and the Degradation of BRD Protein Depends on the Proteasome

**Figure 5:**
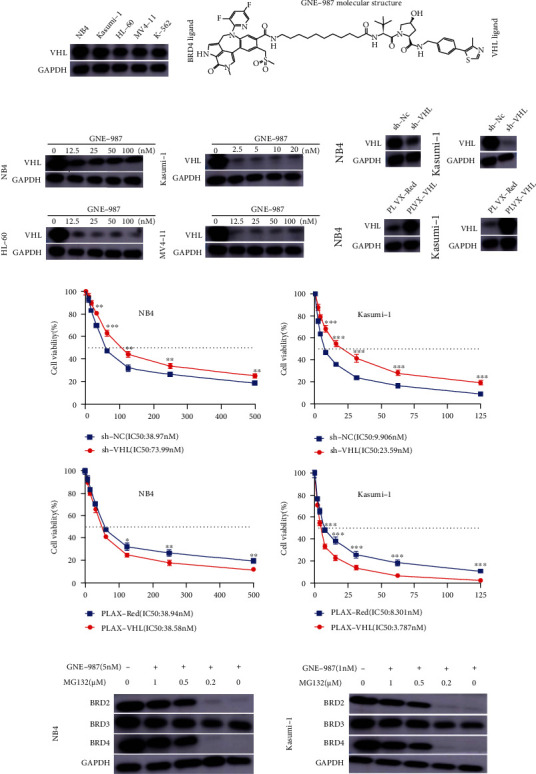
VHL was a powerful helper for GNE-987 in AML cells; BRD protein degradation is proteasome-dependent. (a) Western blot analysis of VHL protein expression in AML cell lines. (b) The molecular structure of GNE-987 contains BRD4 ligand and VHL ligands. (c) Western blot analysis showed that VHL degradation increased after adding different concentration gradients of GNE-987. (d) Knockdown/overexpression of VHL expression by sh-VHL lentivirus/pLX304-VHL-V5 for 5 days in NB4 and Kasumi-1 cells. (e) VHL downregulation increased the half-inhibitory concentration of GNE-987 in NB4 and Kasumi-1 cells, whereas VHL overexpression decreased the half-inhibitory concentrations of GNE-987 in these cells. (f) NB4 and Kasumi-1 cells were treated with GNE-987 and different concentrations of MG132. After treatment for 24 h, BRD2, BRD3, and BRD4 protein levels were investigated by Western blotting analysis.

VHL is a powerful assistant of GNE-987. We tested the expression of VHL in AML cell lines. VHL was widely expressed in myeloid leukemia cell lines (Figure 5(a)). The molecular structure of GNE-987 contained BRD4 ligand and VHL ligands (Figure 5(b)). After adding different concentrations of GNE-987 to NB4, Kasumi-1, HL-60, and MV4-11 cell lines, the expression of VHL was also significantly reduced when BET was consumed (Figure 5(c)). In addition, we successfully transfected VHL knockdown and VHL overexpression vectors into NB4 and Kasumi-1 cells and verified their expression by Western blotting analysis (Figure 5(d)). GNE-987 could recruit VHL. To illustrate this hypothesis, we conducted the following experiment. VHL downregulation increased the half-inhibitory concentration of GNE-987 in NB4 and Kasumi-1 cells whereas VHL overexpression decreased the half-inhibitory concentration of GNE-987 in these cells. This showed that VHL was the key E3 enzyme for the functioning of GNE987 (Figure 5(e)). To determine the role of the proteasome in GNE-987-induced BRD degradation, we used the proteasome inhibitor MG132 to evaluate proteasome activity (Figure 5(f)). MG132 is widely used to inhibit proteasome activity. The results showed that by blocking the proteasome with MG132, BRD proteins increased in a dose-dependent manner. In summary, these data indicated that GNE-987 induced growth inhibition through a VHL-mediated mechanism.

### 3.6. GNE-987 Has a Strong Antitumor Effect on Patients with Primary AML

**Figure 6:**
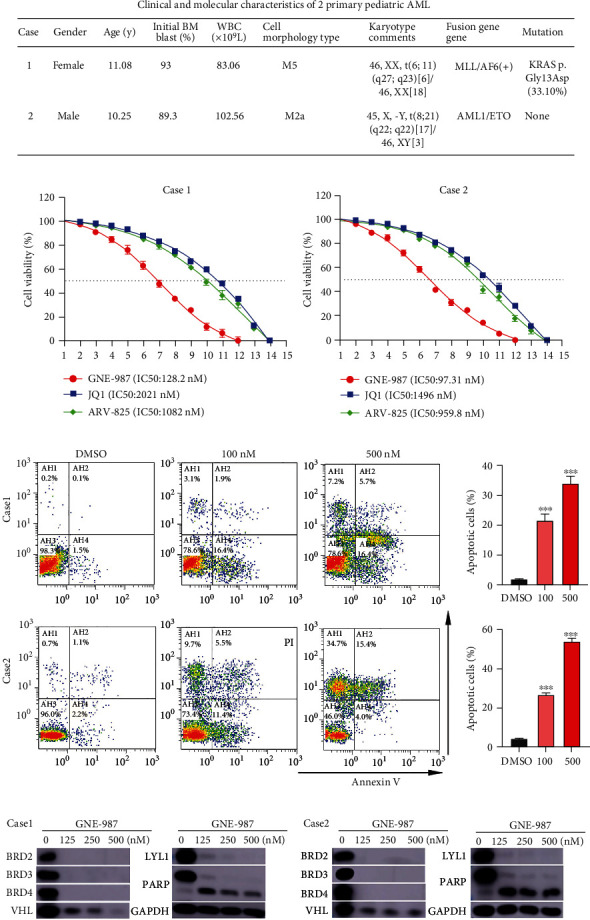
GNE-987 shows cytotoxicity in primary AML cells. (a) Clinical and molecular characteristics of 2 primary pediatric AML. (b) The drug sensitivity of 2 cases of primary cells treated with gradient concentrations of GNE-987, JQ1, and ARV-825 for 24 hours was determined. (c) After 24 hours of treatment with DMSO or different concentrations of GNE-987, the primary cells were analyzed for Annexin V and PI-labeled apoptosis by flow cytometry. The apoptotic rate of primary cells increased significantly after GNE-987 treatment. (d) The Western blot analysis showed that GNE-987 induced the degradation of BET protein, and VHL and LYL1 proteins were also downregulated, and PARP in these primary AML cells increased.

We used two diagnostic AML samples from children to determine whether the primary pediatric AML cells are sensitive to GNE-987 treatment. The clinical and molecular characteristics of 2 cases of primary AML in children are shown in Figure 6(a). Consistent with the results of the previous AML cell lines, the sensitivity of primary cells to GNE-987 was much higher than that of JQ1 and ARV-825 (Figure 6(b)). We treated primary cells with DMSO or different doses of GNE-987 and found that GNE-987 promoted cell apoptosis (Figure 6(c)). In GNE-987-treated primary AML cells, the expression levels of BRD2, BRD3, BRD4, and VHL proteins also decreased, and PARP increased, which was consistent with the results of the cell line. In GNE-987-treated cultures, LYL1 protein levels were also significantly reduced (Figure 6(d)).

### 3.7. In Vivo Studies Confirm That GNE-987 Has a Powerful Antitumor Effect

**Figure 7:**
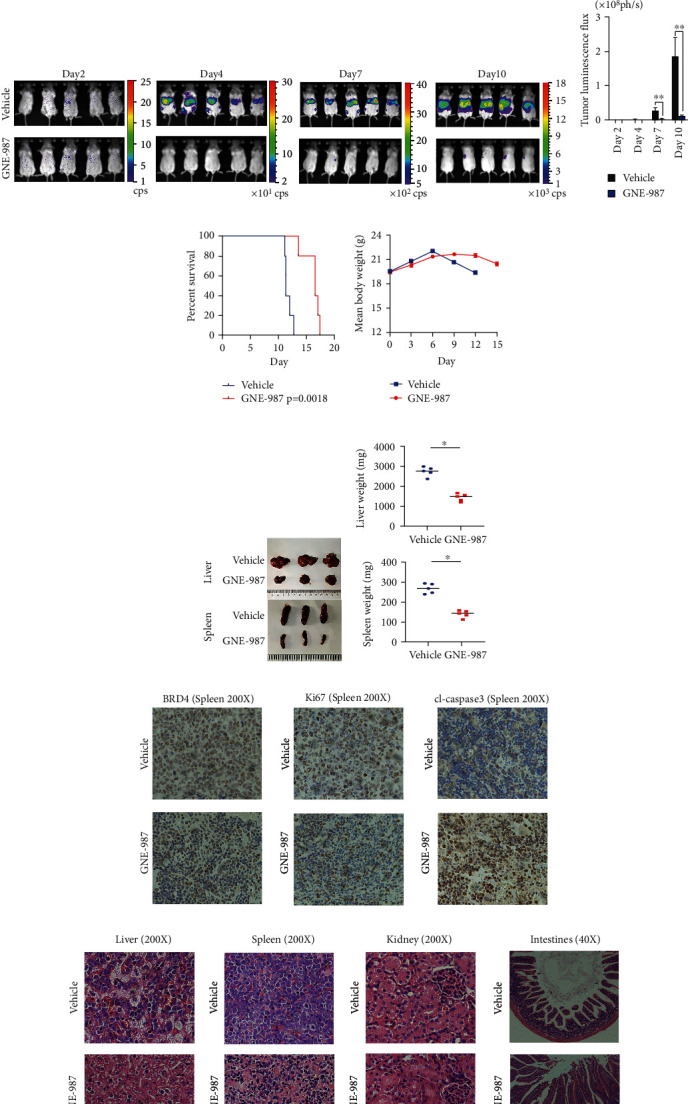
In vivo studies confirm that GNE-987 has a powerful antitumor effect. (a) Schematic diagram of the in vivo experiment. (b) On days 2, 4, 7, and 10, we used the NightOWL In Vivo Imaging System to image each group of mice. (c) The comparison of the fluorescence statistics of the two groups of mice shows that GNE-987 can significantly reduce liver and spleen infiltration in mice. (d) GNE-987 can extend the survival time of mice. (e) The body weights of the two groups were not significantly different. (f) Photographs of the liver and spleen of two groups of mice. (g) Liver and spleen weight from GNE-987- or vehicle-treated mice. (h) IHC staining of BRD4, Ki67, and cleaved-caspase 3 in AML xenograft models from GNE-987- or vehicle-treated mice. (i) HE-stained sections of the liver, spleen, kidney, and intestine from GNE-987- or vehicle-treated mice.

To further examine the in vivo activity of GNE-987, we used P388-D1 cells to establish a preclinical model of AML. The entire operation process is shown in Figure 7(a). On days 2, 4, 7, and 10, we used the NightOWL In Vivo Imaging System to image each group of mice (Figure 7(b)). Compared with the control group, the liver and spleen infiltration of the mice in the GNE-987 treatment group was significantly reduced. The histogram of tumor luminous flux showed that the GNE-987 group was much lower than the control group (Figure 7(c)). By comparing the survival time of the two groups of mice, it is proved that GNE-987 could prolong the lifespan of mice (Figure 7(d)). The difference in the body weights of the two groups was not significant, indicating that GNE-987 had no obvious side effects (Figure 7(e)). The mice were dissected to obtain liver and spleen specimens. The size and weight of the liver and spleen of the GNE-987 treatment group were significantly smaller than those of the control group (Figures 7(f) and 7(g)). In the spleen of the GNE-987 treatment group, BRD4- and Ki67-positive cells decreased, whereas the proportion of cleaved-caspase 3-positive cells in the spleen of the GNE-987 treatment group increased (Figure 7(h)). The HE-stained sections of the liver, spleen, kidney, and intestine of the two groups of mice showed that the tumor cells of the liver and spleen were significantly reduced after GNE-987 treatment, the pathological changes in the kidney were not obvious, and intestinal injury was mild, which also indicated that GNE-987 did less damage to the organs (Figure 7(i)).

### 3.8. GNE987 Treatment Downregulated the Expression of Super-Enhancer-Related Gene LYL1 in AML Cells

**Figure 8:**
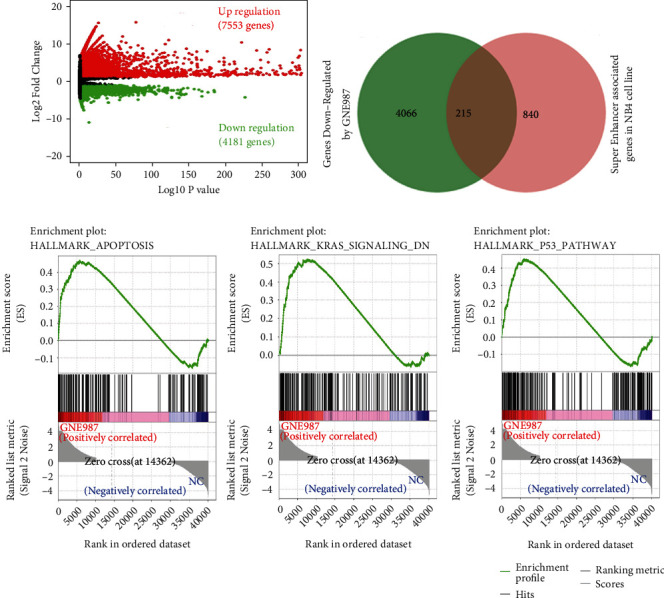
In AML cells, after GNE987 treatment, the expression of many super-enhancer-related genes is downregulated. (a) Compared with the control group, in GNE987-treated NB4 cells, the expression of a total of 11834 genes was affected (upregulation of 7553 genes and downregulation of 4281 genes). (b) Venn diagram of genes related to superenhancers and sensitive to GNE987 in NB4 cells. (c) The GSEA diagrams show that the differently expressed genes in GNE987-treated NB4 cells were enriched in the apoptosis, KRAS, and P53 signaling pathways.

**Figure 9:**
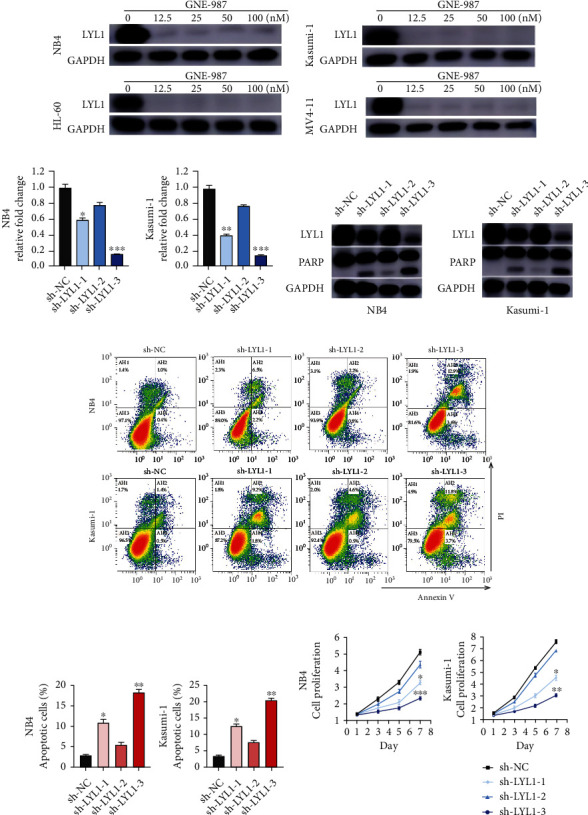
LYL1 is necessary for AML cell growth and survival. (a) Western blot analysis showed that after adding different concentration gradients of GNE-987 to the AML cell lines, LYL1 downregulation increased. (b) Detection of LYL1 knockdown efficiency in NB4 and Kasumi-1 cell lines by qPCR. (c) Detection of LYL1 knockdown efficiency in NB4 and Kasumi-1 cell lines by Western blotting. (d) Flow cytometry showed that knockdown LYL1 increased the apoptosis rate of NB4 cells and Kasumi-1 cells. (e) Statistical histogram of the apoptosis rate of different sh-LYL1 sequences. (f) Knockdown of LYL1 can inhibit the proliferation rate of NB4 cells and Kasumi-1 cells.

We performed RNA-seq gene expression profile analysis on NB4 cells after GNE987 treatment. Compared with the DMSO-treated control group, in GNE987-treated NB4 cells, the expression of 7553 genes was upregulated and the expression of 4281 genes was downregulated (Figure 8(a)). The GSEA diagram showed that the differentially expressed genes were enriched in apoptosis, KRAS, and P53 signaling pathways (Figure 8(c)). Then, we combined ChIP-seq superenhancer profiling and gene expression analysis to determine the key oncogenes involved in the pathogenesis of AML. We performed H3K27ac ChIP-seq detection in NB4 cells and filtered 215 super-enhancer-related genes in NB4 cells which were also downregulated in GNE987-treated NB4 cells (Figure 8(b)). LYL1 is involved in the 215 genes. Essentially, after AML cell lines were treated with GNE987, the expression of the super-enhancer-related gene LYL1 was also significantly downregulated (Figure 9(a)). We detected the LYL1 knockdown efficiency in the NB4 cell line and Kasumi-1 cell line by qPCR (quantitative PCR) and Western blotting (Figures 9(b) and 9(c)). Consistent with the expected results, after we downregulated LYL1, we observed significant apoptosis induction in NB4 and Kasumi-1 cell lines. Among them, sh-LYL1-3-induced apoptosis was the most obvious, and the difference was statistically significant (Figures 9(d) and 9(e)). In addition, gene knockout analysis showed that the downregulation of LYL1 significantly inhibited the growth of NB4 and Kasumi-1 cell lines (Figure 9(f)).

## 4. Discussion

AML is a relatively common malignant tumor in children with poor prognosis and complex etiology [22]. It is necessary to understand its pathogenesis in detail to improve the treatment and prognosis of AML [23]. BRD4 plays an important role in multiple cancer types, such as prostate cancer, lung cancer, and hematological malignancies [24–26]. However, the biological significance of BRD4 in AML is unclear. We found that in CCLE (Cancer Cell Line Encyclopedia) samples, BRD4 was the top gene in AML cell lines compared to other types of cancer cell lines. RNA-seq analysis in this study showed that AML samples had higher BRD4 mRNA expression levels than healthy samples; moreover, BRD4 contributed to a worse prognosis in children with AML. Studies have shown that BRD4 can accumulate in superenhancer regions involved in the control of key oncogenes, such as c-Myc, Bcl-xl, and Bcl-2 [15, 24–26]. This suggests that it can serve as a novel therapeutic target to improve prognosis.

Many BRD4 inhibitors, such as JQ1 and OTX015, have obvious drawbacks. For example, these drugs can only inhibit the growth of a few tumor cells in patients with stage I, promote cell apoptosis, and cannot continuously inhibit transcription [2, 15]. Therefore, new ideas for improving BRD4 inhibition are urgently needed, and GNE-987 came into being. GNE-987 is designed to be an irreversible covalent inhibitor that can achieve the desired effect at a lower drug concentration. The ternary complexes of BRD4B1 or BRD4B2 and VHL promoted by PROTAC GNE-987 were determined by high-resolution natural mass spectrometry. Moreover, the ternary complex of BRD4B1 forms is larger than the ternary complex containing BRD4B2, which indicates that the complex containing BRD4B1 is more stable [27]. Through the direct measurement of natural mass spectrometry, we understood the relationship between the ligase and the PROTAC target, the number of ternary complexes formed, and the balance between the binary and ternary interactions that drive the “hook effect” [28].

As a new type of BRD4 degradation agent, we found that the IC50 of GNE-987 in AML cell lines was less than 100 nmol, which was significantly lower than that of JQ1 and ARV-825. Our study results reveal that GNE-987 can significantly reduce the growth of AML cells in vivo and in vitro by slowing down cell proliferation, interfering with the cell cycle, and accelerating cell apoptosis. These results are consistent with the observations of BRD4 inhibitors such as JQ1 and ARV-825 on solid tumors and AML [29–31], and GNE-987 has more advantages than JQ1 and ARV-825 [32, 33]. BET proteins, including BRD2, BRD3, and BRD4, are epigenome readers known to be associated with acetylated chromatin and transcriptional regulation. Our study found that after treatment with GNE-987, not only are BRD4 degraded but also the levels of BRD2 and BRD3 proteins are reduced, which is similar to the results of other BET inhibitors [15]. Given the high homology between BET family members, GNE-987 can bind to all BET family members. GNE-987 is a PROTAC targeting BRD4 and BET proteins. It can undergo VHL-mediated proteasome degradation and can greatly consume BET protein. This can describe the discovery of a new and highly active chimeric BET degradation agent, which contains an effective BET binder/inhibitor, a VHL-binding fragment, and a ten methylene spacer. VHL targets the chimeric BET degrading agent payload to deliver to the tumor, which may explain the potent killing effect of GNE-987 on AML.

At present, superenhancers are a hot spot in tumor research. Compared with ordinary enhancers, superenhancers can recruit numerous transcription/cofactors and induce the transcription of many target genes. Studies have shown that superenhancers are closely related to oncogenes [34]. However, the biological significance of superenhancers in AML is unclear, so it is urgent to study the key superenhancers in AML. In this study, we found that adding GNE-987 to AML cells could downregulate the expression of super-enhancer-related genes, including LYL1. Previous studies have shown that LYL1 can play a role in renal clear cell carcinoma and osteosarcoma, and copy number amplification occurs in glioma [35–37]. A study also shows that the expression of LYL1 in AML is higher than that in normal bone marrow [9]. LYL1 plays a role in the seven transcription factors of human CD34+ hematopoietic stem progenitor cells (hsps), and LYL1 can also affect the prognosis of AML [38]. Generally, in our study, while GNE-987 consumes BET in AML cell lines, it also downregulated the expression of multiple super-enhancer-related genes, including LYL1. These findings provide new insights into the pathophysiology of AML and provide a new direction for the treatment of AML.

The well-studied JQ1 (BRD4 inhibitor), THZ1 (CDK7 inhibitor), and CBP30 (EP300 inhibitor) have been tested in clinical trials. For example, alvocidib (NCT03298984, NCT03969420, and NCT02520011) listed by Tolero Pharmaceuticals as a cyclin-dependent kinase inhibitor has entered phase II trials in AML and has been reported to have significant activity in patients with relapsed or refractory AML [39]. In the present study, we describe a potent transcriptional interfering factor, the BRD4-targeting inhibitor GNE-987. It has strong antitumor activity in AML cell lines, primary child AML samples, and in vivo experiments. The clinical value of GNE-987 in the treatment of AML needs to be evaluated in future studies.

## 5. Conclusions

In summary, the results of this study show that GNE-987 has strong antitumor activity in AML cell lines, primary child AML samples, and in vivo experiments. GNE-987 exerts its antitumor effect by degrading BET protein, and VHL is the key E3 enzyme for GNE-987 functioning. In addition, GNE-987 can also downregulate the expression of super-enhancer-related genes in AML cells, including the expression of LYL1, which is closely related to AML. These results indicate that GNE-987 may be a promising treatment for AML and is worthy of further investigation.

## Figures and Tables

**Table 1:** shRNAs used to knock down LYL1.

Name	Sequence
Homo-LYL1-sh1	CCGGAGAAGGCAGAGATGGTGTGTGCTCGAGCACACACCATCTCTGCCTTCTTTTTTTGAATT
Homo-LYL1-sh2	CCGGCACTTTGGCCCTGCACTACCACTCGAGTGGTAGTGCAGGGCCAAAGTGTTTTTTGAATT
Homo-LYL1-sh3	CCGGCTTCCTCAACAGTGTCTACATCTCGAGATGTAGACACTGTTGAGGAAGTTTTTGAATT

**Table 2:** Primers used for qRT-PCR analyses.

Name	Sequence (5′⟶3′)
LYL1 forward	ACAGTGTCTACATTGGGCCAG
LYL1 reverse	GGCTGCTAGGGAAGATGCT
GAPDH forward	ACAACTTTGGTATCGTGGAAGG
GAPDH reverse	GCCATCACGCCACAGTTTC

## Data Availability

The data used and/or analyzed during the current study are available from the corresponding authors on reasonable request (GSE188750 and GSE188891).

## Abbreviations

AML: Acute myeloid leukemia
BRD2/3/4: Bromodomain containing 2/3/4
PROTAC: Proteolysis targeting chimeras
PARP: Poly ADP-ribose polymerase
RNA-seq: RNA sequencing
ChIP-seq: Chromatin immunoprecipitation sequencing
VHL: Von Hippel Lindau
SE: Superenhancer
LYL1: Lymphoblastic leukemia 1
shRNA: Short-hairpin RNA
BET: Bromodomain and extraterminal
BETP: Bromodomain and extraterminal protein
HLH: Helix loop helix
TF: Transcription factor
Tal1: T-cell acute lymphoblastic leukemia 1
SCL: Stem cell leukemia
TCL5: T-cell leukemia/lymphoma 5
Tal2: T-cell acute lymphoblastic leukemia 2
MYC: Myelocytomatosis viral oncogene
BRD4B1 and BRD4B2: BRD4 bromodomains 1 and 2
SPR: Surface plasmon resonance
RPMI1640: Roswell Park Memorial Institute 1640
FBS: Fetal bovine serum
DMSO: Dimethyl sulfoxide
CCK8: Cell Counting Kit 8
PBS: Phosphate-buffered saline
PCR: Polymerase chain reaction
GAPDH: Glyceraldehyde 3-phosphate dehydrogenase
HE: Hematoxylin and eosin
HISAT: Hierarchical Indexing for Spliced Alignment of Transcripts
GEPIA: Gene Expression Profiling Interactive Analysis
qPCR: Quantitative PCR
CCLE: Cancer Cell Line Encyclopedia.
